# SiR‐XActin: A Fluorescent Probe for Imaging Actin Dynamics in Live Cells

**DOI:** 10.1002/anie.202509285

**Published:** 2025-10-16

**Authors:** Veselin Nasufovic, Julian Kompa, Halli L. Lindamood, Merle Blümke, Rayane Dibsy, Birgit Koch, Victoria Levario‐Diaz, Katharina Weber, Marlene Maager, Ekaterina Nomerotskaia, Arnaud Echard, Elisabetta Ada Cavalcanti‐Adam, Eric A. Vitriol, Hans‐Dieter Arndt, Kai Johnsson

**Affiliations:** ^1^ Institut für Organische und Makromolekulare Chemie Friedrich‐Schiller‐Universität Humboldtstr. 10 D‐07743 Jena Germany; ^2^ Department of Chemical Biology Max Planck Institute for Medical Research Jahnstrasse 29 D‐69120 Heidelberg Germany; ^3^ Institute of Chemical Sciences and Engineering École Polytechnique Fédérale de Lausanne (EPFL) CH A3 398 Station 6 Lausanne CH‐1015 Switzerland; ^4^ Department of Neuroscience and Regenerative Medicine Medical College of Georgia at Augusta University 1120 15th Street Augusta Georgia 30912 USA; ^5^ Department of Cellular Biophysics Max Planck Institute for Medical Research Jahnstrasse 29 D‐69120 Heidelberg Germany; ^6^ Chair of Cellular Biomechanics University of Bayreuth Universitätsstraße 30 D‐95447 Bayreuth Germany; ^7^ Membrane, Cytoskeleton and Cell Division Unit CNRS UMR3691 Institut Pasteur Université Paris Cité 25–28 rue du Dr Roux Paris F‐75015 France

**Keywords:** F‐actin, Fluorescent probes, Jasplakinolide derivatives, No‐wash live‐cell imaging, STED microscopy

## Abstract

Imaging actin‐dependent processes in live cells is important for understanding numerous biological processes. However, currently used natural‐product‐based fluorescent probes for actin filaments affect the dynamics of actin polymerization and can induce undesired cellular phenotypes. Here, we introduce SiR‐XActin, a simplified jasplakinolide‐based, far‐red fluorescent probe that enables bright and photostable staining in various cell types without requiring genetic modifications. Due to its relatively weak binding affinity, the probe exhibits minimal cytotoxicity and labels actin filaments without significantly altering actin dynamics. Furthermore, SiR‐XActin is suitable for time‐resolved, live‐cell super‐resolution STED microscopy. Exchanging the SiR fluorophore in SiR‐XActin for other fluorophores yields probes in different colors. All these properties make SiR‐XActin and its analogs powerful tools for studying actin dynamics using live‐cell fluorescence microscopy.

## Graphical abstract

SiR‐XActin is a far‐red fluorescent probe for live‐cell imaging of actin that avoids genetic modification and minimizes disruption of actin dynamics, due to its lower binding affinity. Based on jasplakinolide‐like natural product hybrids and Si–Rhodamine, it offers photostable live‐cell no‐wash staining across cell types and microscopy techniques. Fluorophore exchange allows multicolor labeling and combination with other probes or chemigenetic tools.

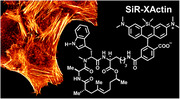

## Introduction

**Figure 1:**
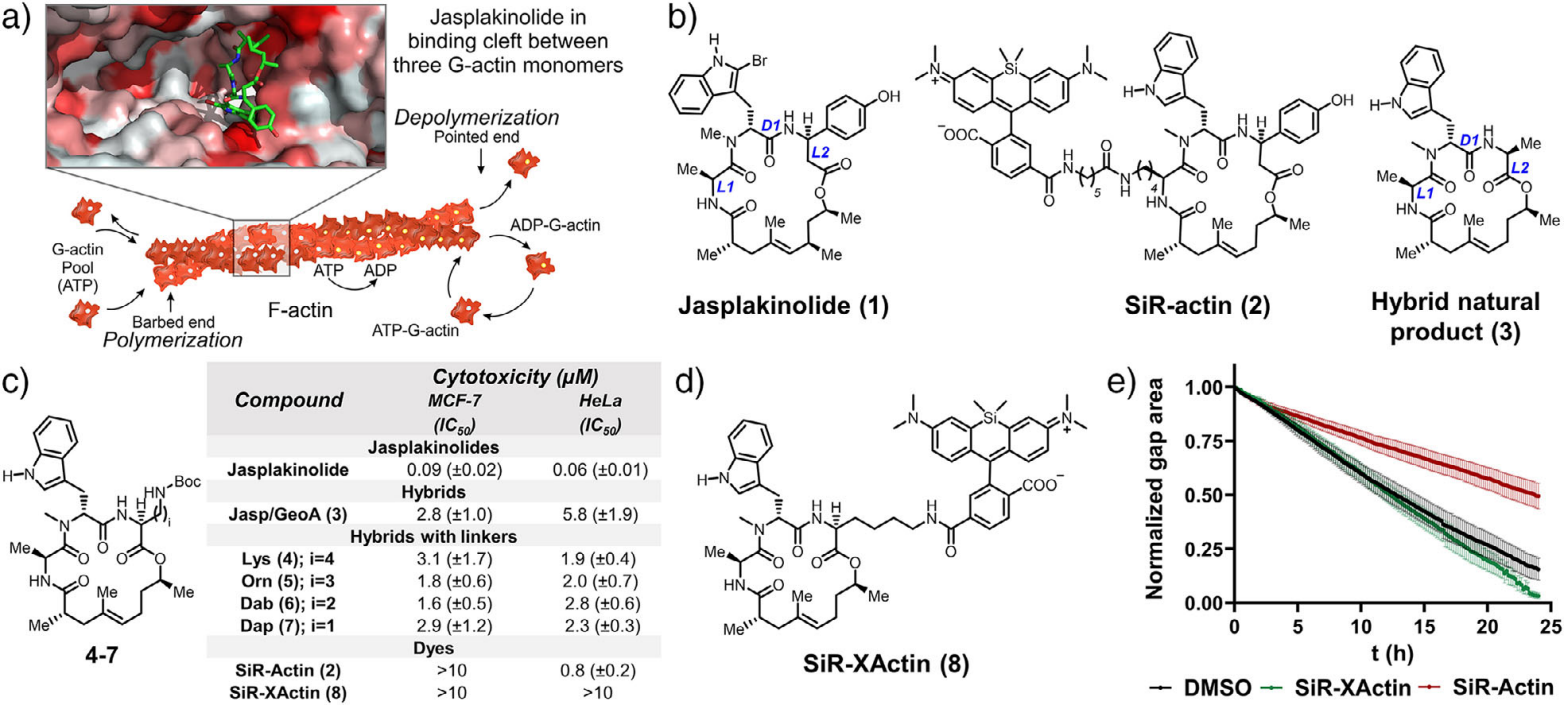
SiR‐XActin is a hybrid natural product with reduced cytotoxicity and perturbation of cellular dynamics: a) Scheme of actin polymerization/depolymerization machinery and binding cleft of jasplakinolide‐like natural products. Cryo‐EM structure of jasplakinolide‐stabilized F‐actin (PDB: 6T24) was used for generating cartoon of jasplakinolide binding to F‐actin. b) Structures of natural product jasplakinolide (**1**), cell permeable F‐actin labeling tool SiR‐actin (**2**), non‐toxic hybrid natural product (**3**). c) Synthesized analogs and cytotoxicity profiling in HeLa (ACC‐57, DSMZ) and MCF‐7 (ACC‐115, DSMZ). d) Structure of SiR‐XActin (**8**). e) Comparison of SiR‐actin and SiR‐XActin influence on migration of HeLa (Kyoto) cells in wound healing assay, 500 nM dye concentration used. Shown curves represent normalized wound gap area over time. Average values and standard deviation of five independent measurements.

The actin network is an essential part of the eukaryotic cytoskeleton and has both structural roles and dynamic functions, for example in cytokinesis, cell‐shape adaptation, cell division, and organelle trafficking.^[^ ^1^ ^]^ Actin is one of the most abundant proteins in eukaryotic cells with a high level of evolutionary sequence and structure preservation.^[^ ^2^ ^]^ Globular actin (G‐actin, 42 kDa) polymerizes in a highly regulated process to form microfilaments (F‐actin).^[^ ^3^, ^4^ ^]^ Numerous actin‐binding proteins (ABPs) interact with G‐actin or F‐actin to regulate its functions (Figure 1a).^[^ ^5^ ^]^ F‐actin is often organized in stable networks (e.g., myofibrils, axon rings) or dynamic higher‐order organizational networks (e.g., filopodia, lamellipodia, actin cortex, contractile rings, focal adhesions, stress fibres).^[^ ^6^ ^]^

An important advance in the visualization of F‐actin in fixed and permeabilized cells was the development of fluorescent derivatives of the F‐actin‐binding natural product phalloidin.^[^ ^7^ ^]^ Phalloidin binds at the interface of three actin monomers in F‐actin, increasing the stability of the fiber and slowing the dynamic actin polymerization/depolymerization process.^[^ ^8^, ^9^, ^10^, ^11^ ^]^ In 2014, derivatives of the natural product jasplakinolide, which competes in binding with phalloidin, were conjugated to the far‐red fluorogenic dye Si–Rhodamine (SiR), resulting in the cell‐permeable SiR‐actin (Figure 1b, compound 2).^[^ ^12^, ^13^, ^14^ ^]^ This, enabled visualization of F‐actin in living cells, such as immortalized cell lines, primary cells, organoids, and tissues.^[^ ^15^, ^16^ ^]^ Subsequently, jasplakinolide derivatives with different fluorophores were introduced to complement SiR‐actin.^[^ ^17^, ^18^, ^19^ ^]^ However, jasplakinolide and its fluorescent derivatives stabilize F‐actin and can affect the dynamics of actin polymerization/depolymerization.^[^ ^20^, ^21^, ^22^ ^]^ More recently, another fluorescent probe, SPY650‐FastAct, was introduced for imaging of dynamic actin populations, however, its structure wasn't revealed.^[^ ^23^ ^]^

Bacterial actin‐binding peptides (LifeAct),^[^ ^24^ ^]^ actin‐binding proteins,^[^ ^25^ ^]^ fluorescent protein fusions with G‐actin^[^ ^26^ ^]^ and de novo engineered small proteins^[^ ^27^, ^28^ ^]^ as fused to fluorescent‐ or to self‐labeling proteins are commonly used as genetically encoded alternatives for imaging F‐actin. In particular, among these, LifeAct has become a popular tool for imaging F‐actin, due to its relatively small effect on the dynamics of actin (de)polymerization, provided its expression levels are strictly controlled.^[^ ^20^, ^21^ ^]^ However, LifeAct requires the genetic modification of cells, making small molecule probes the preferred and often the only option for many applications.

Here, we introduce new fluorescent simplified jasplakinolide derivatives that distinguish themselves by their reduced affinity for F‐actin and associated low cytotoxicity. As a result, these probes enable the imaging of highly dynamic F‐actin populations in live cells.

## Results and Discussion

We have recently shown that a hybrid of natural products jasplakinolide and geodiamolide A, compound **3** (Figure 1b), exhibits only weak cytotoxicity in comparison to parental jasplakinolide **1**.^[^ ^29^ ^]^ Most cytotoxic jasplakinolide‐like natural products have a defined combination of three amino acids (L1‐, D1‐, and L2, Figure 1b), with L2 being a β‐L‐amino acid with an aromatic side chain (most often tyrosine) when D1 is D‐tryptophan. We demonstrated that a hitherto non‐reported combination in isolated natural products (D‐tryptophan as D1 and an α‐amino acid as L2) binds actin fibers (e.g., compound **3**, Figure 1b), but does not influence cell viability significantly.^[^ ^29^ ^]^

Based on this finding, we reasoned that such a ligand would be an excellent starting point for the development of imaging probes with reduced toxicity. To convert **3** into a scaffold for fluorescent F‐actin probes, we synthesized a series of jasplakinolide derivatives with different linker structures (Figure 1c, Scheme SI1–3). These analogs showed similar low cytotoxicity as **3** and we chose analog **4** (named XActin) bearing a lysine linker for attachment of fluorescent probes. Cryo‐EM structures of jasplakinolide and its analogs binding to F‐actin, as well as docking of possible conjugates, indicate that this lysine residue is pointing outside the binding cleft and should allow the attachment of fluorophores without disturbing the binding significantly (Figures 1a and SI1).^[^ ^30^, ^31^, ^32^ ^]^ Attaching Si–rhodamine (SiR) to XActin yielded the far‐red fluorescent conjugate SiR‐XActin (Figure 1d, Scheme SI4). SiR‐XActin showed no significant cytotoxicity in HeLa (ACC‐57) cells at all concentrations tested (IC_50_ > 10 µM), whereas SiR‐actin showed sub‐micromolar toxicity under these conditions (Figures 1d and SI2). Interestingly, neither SiR‐actin nor SiR‐XActin influenced cell viability of MCF‐7 (ACC‐115) cell line (Figures 1d and SI2). Since inhibition of actin dynamics directly effects cell motility,^[^ ^33^ ^]^ we compared the behavior of SiR‐actin and SiR‐XActin in a wound healing assay. Whereas SiR‐XActin showed no significant influence on the migration of HeLa (Kyoto) cells and gap closure, SiR‐actin significantly slowed down cell motility, preventing wound fronts from closing the gap (Figures 1e and SI3).

**Figure 2:**
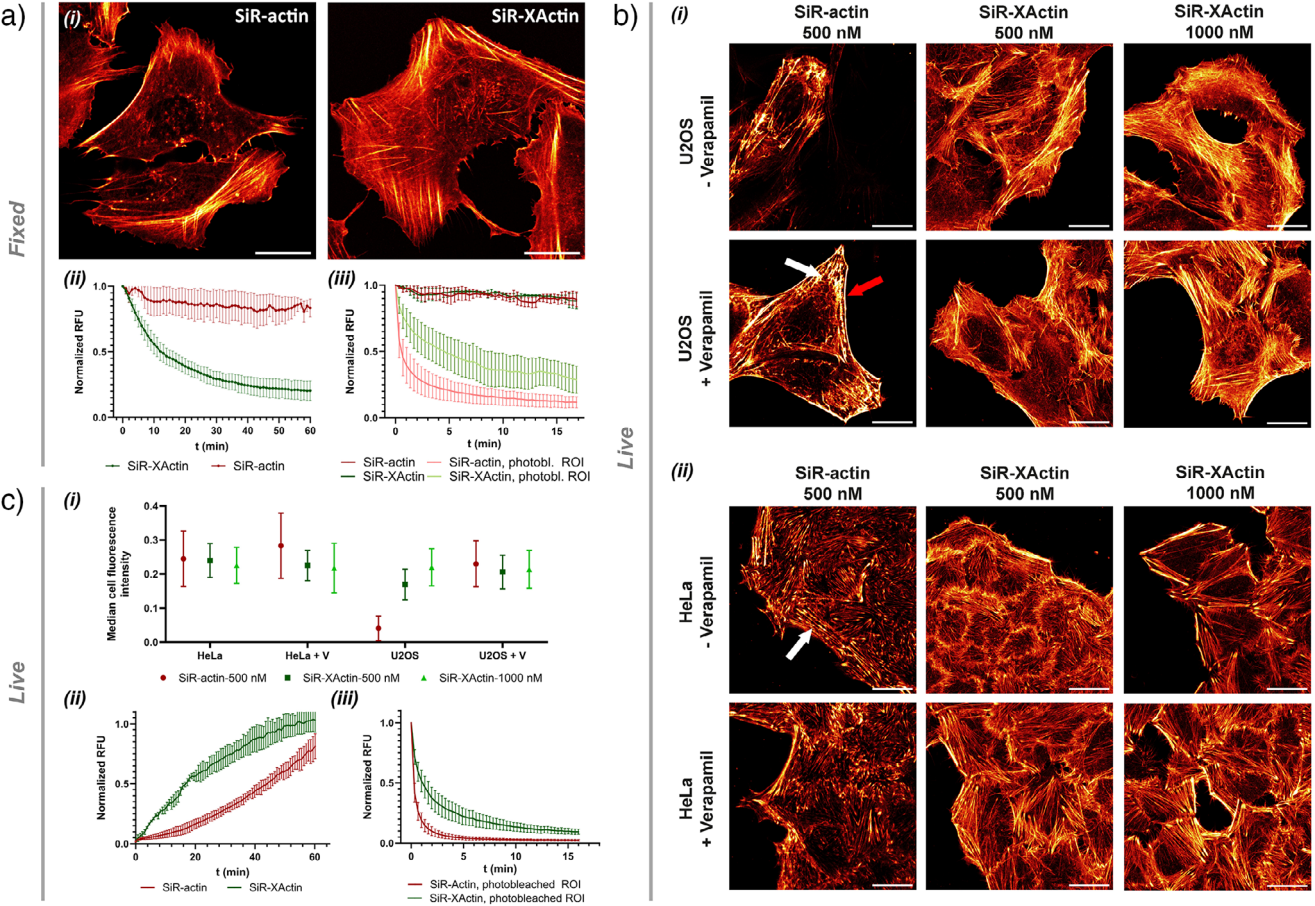
Labeling performance of SiR‐XActin in fixed and live cells: a) *i*: Fixed‐cell no‐wash CLSM images of U‐2 OS cells. 652 nm excitation with 0.4% (SiR‐actin) or 2% (SiR‐XActin) laser power. *ii*: Fluorescence signal reduction in fixed U‐2 OS cells labeled with 500 nM corresponding dye after medium exchange. The signal was normalized to the no‐wash fluorescence intensity of each replicate. Data from *N* = 3 independent experiments, error bars represent SEM. *iii*: Fluorescence intensity bleaching curve of SiR‐actin and SiR‐XActin (500 nM) in U‐2 OS cells. 652 nm excitation (0.1% laser power) used for imaging, 652 nm (100% laser power) used for bleaching. The signal was normalized to the initial fluorescence intensity of each measurement. Data from *N* = 3 independent experiments, error bars represent SEM. b) *i*: U‐2 OS and *ii*: HeLa (Kyoto) cells stained with SiR‐actin and SiR‐XActin at the given dye concentrations in presence and absence of verapamil (10 µM), 4 h of incubation. c) *i*: Median fluorescence signal in U‐2 OS and HeLa (Kyoto) cells (condition described in B‐*i* and B*‐ii*, V–verapamil). Data from *N* = 3 independent experiments, error bars represent SD. *ii*: Live‐cell labeling kinetics of SiR‐actin and SiR‐XActin (both 500 nM) in HeLa (Kyoto) cells, signal was normalized to signal intensity measured after 4 h of incubation. Data from *N* = 3 independent experiments, error bars represent SD. *iii*: Live‐cell bleaching curves for SiR‐actin and SiR‐XActin (both 500 nM) in HeLa (Kyoto) cells, incubation time 4 h, 652 nm excitation (0.1% laser power) used for imaging, 652 nm (100% laser power) used for bleaching, imaging–bleaching cycle every 20 s. Red Hot lookup–pixel intensities were scaled between 0 (black) and 65535 (bright yellow) by Fiji. Scale bars 20 µm.

When incubated with fixed cells, SiR‐XActin showed a typical F‐actin staining profile by confocal laser‐scanning microscopy (CLSM), comparable with SiR‐actin (Figures 2a‐*i* and SI4). However, when the medium was exchanged for one without the respective probe, the specific signal from SiR‐XActin staining was quickly washed out, in contrast to the signal of SiR‐actin, which remained stable (Figure 2a‐*ii*) and only competition with high concentration of jasplakinolide outcompetes its binding significantly (Figure SI4). We then measured the apparent affinities of SiR‐XActin and SiR‐actin for F‐actin in a microscopy‐based assay. SiR‐actin showed a *K*d_app_ of 6.0 (±0.3) nM, which is in agreement with a previously reported value,^[^ ^34^ ^]^ whereas SiR‐XActin showed an approximately 100‐fold lower affinity with a *K*d_app_ of 557 (±266) nM. These differences in binding affinities (Figure SI5) provide a rationale for the striking differences in cytotoxicity as well as in cell motility.

It has been previously demonstrated that weak‐affinity fluorescent probes provide higher photostability, as the low affinity facilitates the exchange of bleached probes.^[^ ^35^ ^]^ Both SiR‐actin and SiR‐XActin show excellent photostability in regular imaging mode when staining fixed actin filaments (650 nm excitation, 0.1% laser power), however, in photobleaching mode (650 nm excitation, 100% laser power) SiR‐XActin shows significantly slower signal decay (Figure 2a‐*iii*). At the same concentration (500 nM) SiR‐actin shows a 1.8‐fold higher fluorescent signal when staining fixed actin filaments than SiR‐XActin (Figure SI5), whereas at concentrations above the respective *K*d_app_, SiR‐actin and SiR‐XActin show almost identical brightness.

Next, we tested the ability of SiR‐XActin to label F‐actin populations in live cells (Figure 2b,c). SiR‐XActin labels efficiently F‐actin in U‐2 OS and HeLa (Kyoto) cells, with and without the use of the efflux pump inhibitor verapamil. The staining profile of SiR‐XActin in live cells is similar to the stained F‐actin networks in fixed cells (Figure 2a‐*i*). However, SiR‐actin showed a higher preference for stress fibers (white arrows, Figure 2b) and for structures around cell edges (red arrow, Figure 2b‐*i*). Additionally, SiR‐actin staining in live cells resulted in uneven, bright fluorescence accumulation along the fibers, which were absent in fixed samples stained either by SiR‐actin or SiR‐XActin as well as absent in live cells stained by SiR‐XActin. These observations suggest that under these conditions SiR‐actin is inducing non‐physiological actin phenotypes. SiR‐actin requires the presence of verapamil for efficient labeling of F‐actin in U‐2 OS cells, whereas SiR‐XActin shows efficient labeling even in the absence of verapamil (Figures 2b‐*i* and c‐*i*). Efficient labeling in the absence of verapamil was demonstrated for SiR‐XActin in two additional cell lines, 3T3 and COS‐7 (Figure SI6). Verapamil inhibits human P‐glycoprotein and multidrug resistance protein 1 (MRP1) among other reported transporters which actively eliminate xenobiotics such as rhodamines from cells.^[^ ^36^, ^37^, ^38^, ^39^ ^]^ Hence, verapamil is often used in combination with SiR‐actin to increase its intracellular concentration and enhance F‐actin labeling. Considering the potential effects on transporters, calcium channels, and downstream signaling pathways, the ability to use SiR‐XActin without the need for verapamil represents a significant advantage.

When HeLa (Kyoto) cells were incubated with a range of concentrations of SiR‐XActin and SiR‐actin (0.04–10 µM) for 6 h, only SiR‐actin induced condensation of F‐actin, similar to that previously reported for other jasplakinolide derivatives.^[^ ^40^ ^]^ SiR‐actin induced the formation of brightly labeled aggregates in concentration ranges of 0.1–10 µM (Figure SI7A). In contrast, such aggregation was not observed with SiR‐XActin, regardless of the concentration used (Figure SI7A). SiR‐actin‐treated (0.1–10 µM) HeLa (Kyoto) cells showed membrane blebbing (Figure SI8), a phenotype that also has been reported previously for jasplakinolide itself.^[^ ^41^ ^]^ Membrane blebbing was not observed with SiR‐XActin even at high concentrations (10 µM) (Figure SI8). In U‐2 OS cells, after 16 h incubation time, neither SiR‐actin nor SiR‐XActin induces blebbing (Figure SI9), but SiR‐actin staining results in bright fluorescence accumulation along stress fibers, even at lower concentrations (Figures SI7B and 2B‐*i*). The above comparisons were done at concentrations of both probes that are 10‐fold above the *K*d_app_ of SiR‐actin. We therefore also compared staining with SiR‐actin at concentrations of 30 nM and SiR‐XActin at 500 nM. After 2 h incubations, SiR‐XActin reached labeling profile which did not change over time (over 8 h). In contrast, SiR‐actin after 2 h showed only weak labeling in comparison, although its preference for stress fibers and focal adhesions can be seen (Figure SI10).

When the kinetics of labeling were recorded in live HeLa (Kyoto) cells, significant differences were observed between SiR‐XActin and SiR‐actin (Figure 2c‐*ii*). SiR‐XActin showed faster labeling (*t*_1/2_ ∼ 20 min) than SiR‐actin (*t*_1/2_ ∼ 111 min), which also displayed a lag phase, suggesting that SiR‐XActin possesses a higher cell permeability than SiR‐actin (Figure SI11, Video SI1‐2). As observed in fixed cells (Figure 2a‐*iii*), SiR‐XActin also displayed higher photostability than SiR‐actin in live cells (Figure 2c‐*iii*). The low cytotoxicity, high brightness, and photostability make SiR‐XActin attractive for long‐term time‐lapse imaging experiments. To demonstrate this, we incubated live HeLa (Kyoto) cells with SiR‐XActin at a concentration above its *K*d_app_ (1 µM) for 24 h, followed by imaging every 30 s. The signal intensity of SiR‐XActin stayed stable over 35 h and no obvious influence on the actin phenotype was observed, i.e., cells moved freely and divided. Multiple lamellipodiae, filopodiae and contractile rings can be seen during the time‐lapse, indicating once again very low perturbance of this highly regulated network (Figures SI12 and SI13, Videos SI3 and SI4).

Next, we focused on mitotic cell shape changes and cytokinesis—the final step of cell division that results in the physical separation of the daughter cells—both of which are critically dependent on actin dynamics. Recording live HeLa (ATCC) cells showed that SiR‐XActin labeled actin with comparable efficiency in all cells (Figure SI14A) and clearly revealed actin enrichment at the equatorial actomyosin ring during cleavage furrow ingression (arrowheads, Figure SI14B), as well at the intercellular bridge connecting the daughter cells in later time points (arrow, Figure SI14B). Incubation with 1 µM SiR‐XActin did not significantly alter any of the key cell division parameters that we quantified: time for mitotic cell rounding (Figure SI15A), metaphase duration (Figure SI15B), time for cleavage furrow ingression (Figure SI15C) and full cytokinesis duration measured (Figure SI15D) as previously described.^[^ ^42^ ^]^ In contrast to SiR‐XActin, incubation with SiR‐actin yielded weaker and heterogeneous labeling among cells, with poor visualization of F‐actin at the cleavage furrow and no detectable labeling in late intercellular bridges (Figure SI14B). While division parameters were not significantly affected (Figures SI15A–D), we noticed that SiR‐actin‐treated cells displayed abnormal blebbing throughout mitotic cell rounding (*t*10–*t*55 min, Figure SI15E), in marked contrast to SiR‐XActin‐treated cells which only blebbed transiently at the polar cortex during anaphase cell elongation, as expected.^[^ ^43^ ^]^ Of note, adding verapamil to SiR‐actin‐treated cells (but not to SiR‐XActin‐treated cells) resulted in the intracellular aggregation of SiR‐actin labeling, extensive blebbing as early as 1 h post‐incubation, even in non‐dividing cells, along with progressive morphological changes indicative of cellular stress and compromised cell health (Figure SI16). In summary, SiR‐XActin enables robust F‐actin staining without affecting mitotic or cytokinesis progression.

**Figure 3:**
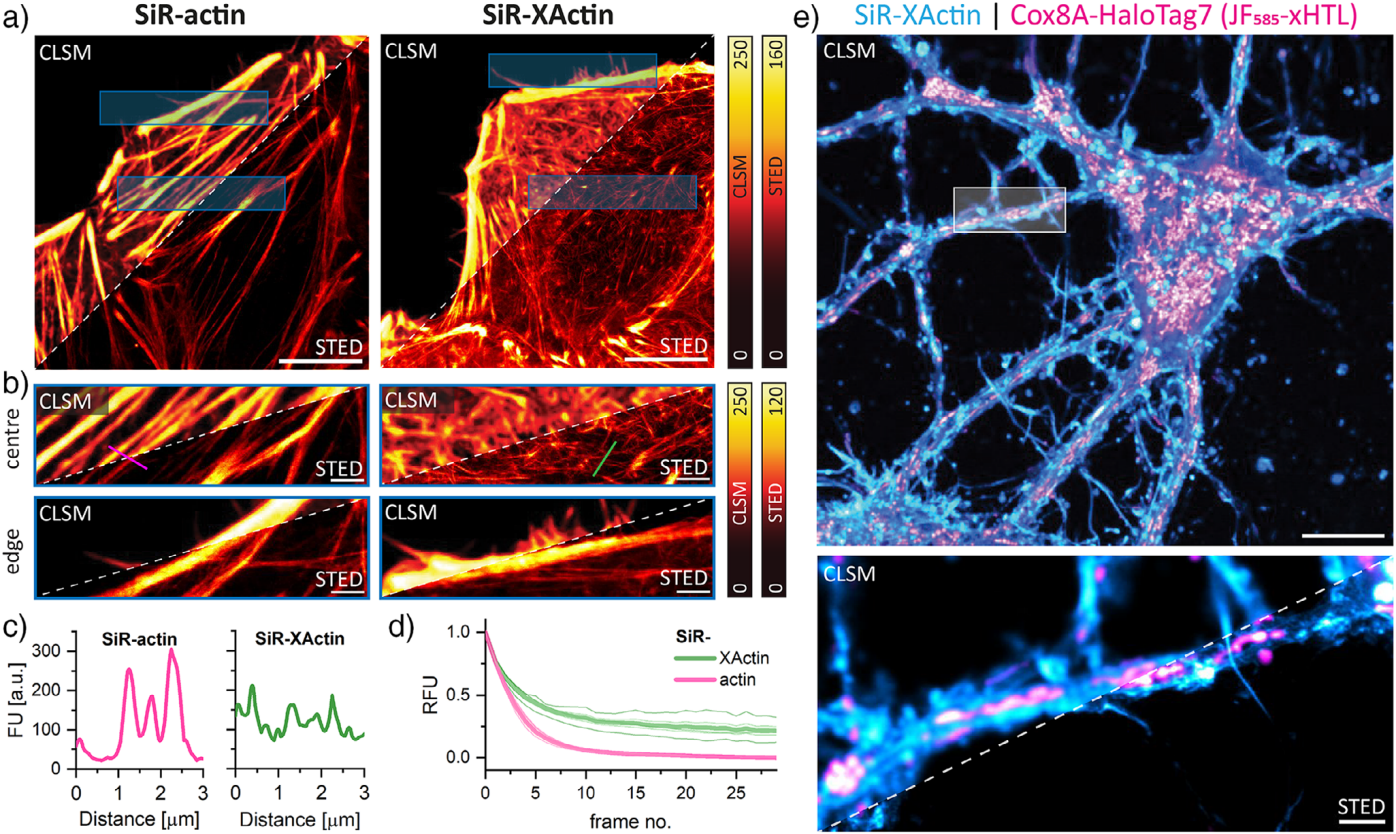
SiR‐XActin is a tool to study F‐actin in live cells by STED microscopy: a) Phenotypic comparison of SiR‐actin and SiR‐XActin stainings of live HeLa (Kyoto) cells. Cells were stained with 1 µM actin probes for 2 h, no‐wash imaging. Representative CLSM and STED images. Pixel intensities were scaled between dark and bright colors according to the reference bars. Scale bars: 10 µm. b) Magnification of blue ROIs from (a). Scale bars: 1 µm. c) Line‐scan profiles shown in (b). (STED) perpendicular to actin filaments. Staining with SiR‐actin reveals increased number and thickness of stress fibers in the center and a fewer filopodia on the cell's edges in comparison to SiR‐XActin staining. d) Normalized fluorescence intensity of multi‐frame STED imaging in a 10 x 10 µm ROI. U‐2 OS cells were stained with 1 µM SiR‐actin or SiR‐XActin supplemented with 10 µM verapamil for 2 h. After an initial bleaching phase, SiR‐XActin reveals improved apparent photostability. e) Combination of SiR‐XActin with exchangeable HaloTag Ligands (xHTL) for multi‐color STED. Hippocampal rat neurons were transduced with rAAVs with TOM20‐HaloTag7 at seven DIV and imaged at twelve DIV (days in vitro). Exchangeable HaloTag Ligand (JF_585_‐S5, 500 nM) and SiR‐XActin (1 µM) were co‐imaged with the same depletion laser (775 nm). Scale bars: 10 µm (overview), 1 µm (magnification).

To demonstrate further usability of SiR‐XActin, we performed dual‐color CLSM imaging together with the focal adhesion marker mCherry‐Vinculin (Figure SI17A) and mEmerald‐Paxilin (Figure SI17B).^[^ ^44^ ^]^ SiR‐actin has been very useful for application in live‐cell stimulated emission depletion (STED) nanoscopy,^[^ ^14^, ^16^ ^]^ and we therefore explored the potential of SiR‐XActin for such applications (Figure 3). STED imaging of single HeLa (Kyoto) cells reveals a higher abundance of actin stress fibers of SiR‐actin in comparison to SiR‐XActin staining (Figure 3a). The images obtained for SiR‐XActin staining are similar to STED imaging of the genetically encoded LifeAct‐HaloTag7 with a SiR‐HaloTag Ligand (Figure SI18). Also, labeling with SiR‐actin increases the thickness of stress fibers in the center of the cells (Figure 3b,c) and the appearance of filopodia on the cell's edges is reduced (Figure 3b). SiR‐XActin showed again higher photostability in time‐lapsed STED imaging (Figure 3d, VideoSI5‐7) compared to SiR‐actin. After an initial bleaching phase, SiR‐XActin shows a stable signal of around 30 ± 3% of its initial intensity. We postulate that this difference is mainly due to the lower binding affinity of SiR‐XActin, which should facilitate the exchange of bleached probe, as demonstrated for other weak‐affinity labels.^[^ ^45^, ^46^ ^]^ SiR‐XActin can be used in combination with other (exchangeable) labeling tools for STED imaging. We used SiR‐XActin to label the F‐actin network and either exchangeable HaloTagLigands (JF_585_‐S5, Cox8A‐HaloTag7)^[^ ^46^ ^]^ (Figures 3e and SI19A) or PK Mito Orange (PKMO)^[^ ^47^ ^]^ (Figure SI19B) to label mitochondria in live hippocampal neurons. Both probes can be imaged with the same depletion laser and are compatible with 3D‐STED imaging (Figure SI19C, Video SI6).

**Figure 4:**
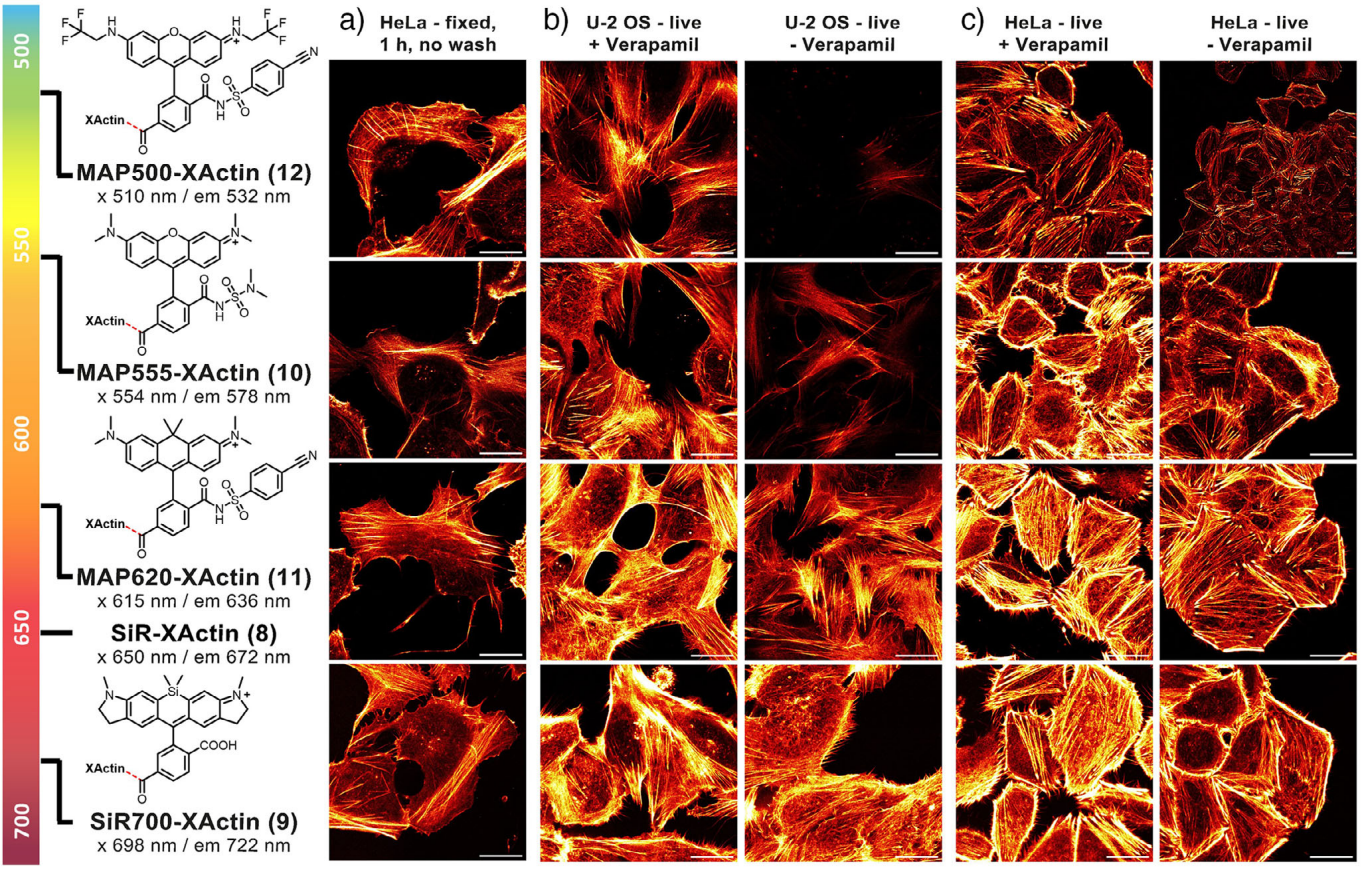
XActin as a conjugate with other fluorophores to cover the whole imaging spectral window: a) Labeling of fixed HeLa (Kyoto) cells, b) labeling of live U‐2 OS cells or c) HeLa (Kyoto) cells with XActin probes, 4 h incubation, with and without 10 µM verapamil. All conditions no‐wash, 500 nM. Laser settings live cells: MAP500‐XActin 0.3%, MAP555‐XActin 0.3%, MAP620‐XActin 0.1%, SiR700‐XActin 0.3% laser power. Two line average. For laser settings in fixed cells see Figure S19. Red hot lookup–pixel intensities were scaled between 0 (black) and 65535 (bright yellow) by Fiji. Scale bars: 20 µm.

XActin ligand **7** can serve as a platform to generate XActin probes with different fluorophores, covering the visible to near‐infrared spectrum (Figures SI20–S21and 4). Conjugation of **7** to the SiR analog Si–Rhodamine700 yielded SiR700‐XActin (**9**), which is further red‐shifted relative to SiR‐XActin with an excitation maximum at 698 nm. SiR700‐XActin showed good labeling properties in fixed and live cells without inducing non‐physiological actin phenotypes (Figures 4 and SI22–S23). Another popular dye for live‐cell imaging is the fluorogenic rhodamine derivative MaP555 (**10**), which has an excitation maximum at 554 nm and thus is suitable for dual‐color imaging with SiR‐based probes using common laser lines.^[^ ^48^ ^]^ In order to further explore the spectral range of fluorogenic rhodamine dyes, we also coupled XActin to MaP derivatives of carbopyronine (MaP620, **11**) and the green rhodamine analog 500R (MaP500, **12**).^[^ ^48^ ^]^ All tested probes showed excellent labeling properties in fixed and live cells, including rat hippocampal neurons, and also showed no change in actin phenotype at the concentrations used for imaging (Figures 4 and SI22–SI23). MaP555‐ and MaP620‐XActin showed satisfying performance in live cell STED imaging (Figure SI24). MaP500‐XActin and MaP555‐XActin stain F‐actin in live U‐2 OS cells better when incubated with verapamil, showing that also the fluorophore itself contributes to efflux (Figure 4).

**Figure 5:**
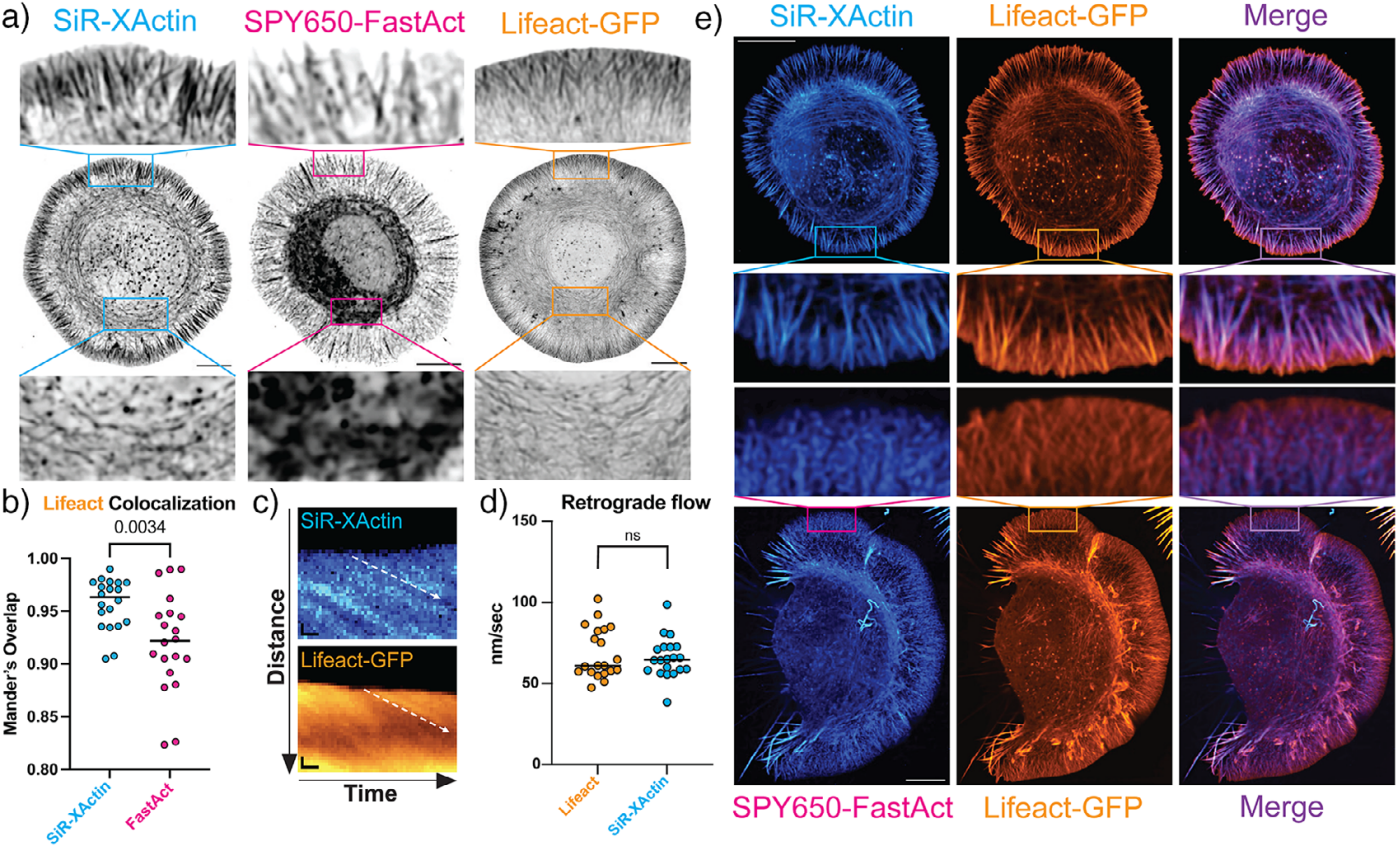
Labeling actin dynamic structures in live CAD cells: a) Representative images of actin imaged with SiR‐XActin, SPY650‐FastAct, or Lifeact‐GFP. Scale bars = 10 µm. b) Colocalization analysis of SiR‐XActin and SPY650‐FastAct with Lifeact‐GFP. *n* = 20 cells, unpaired t‐test. c) Representative kymographs of actin imaged with SiR‐XActin and Lifeact‐GFP. Scale bars, vertical = 1 µm and scale bars, horizontal = 10 s. d) Scatter plot of retrograde flow measurements of Lifeact‐GFP and SiR‐XActin. Each point represents the average measurement of 10 kymographs from each cell. n.s. = not significant, Mann–Whitney test. e) Representative images of cells containing both Lifeact‐GFP and SiR‐XActin (above) or SPY650‐FastAct (below). Scale bar = 10 µm.

Next, we investigated the potential of SiR‐XActin to image fast actin dynamics as they are observed at the cell leading edge in Cath.a‐differentiated (CAD) cells, where the organization of actin, rate of filament assembly, and retrograde flow have been well documented.^[^ ^49^, ^50^, ^51^ ^]^ When compared to LifeAct‐GFP, a standard tool for visualizing actin in live cells that faithfully recognizes most of the polymerized actin structures in CAD cells,^[^ ^52^ ^]^ SiR‐XActin showed similar labeling of actin filaments at the rapidly assembling leading edge and was remarkably improved at visualizing these structures compared to the synthetic probe SPY‐650 FastAct (Figure 5a, Video SI7). This was verified by measuring the Mander's overlap coefficient in colocalization experiments where SiR‐XActin and SPY‐650 FastAct were used in cells already expressing LifeAct‐GFP (Figure 5b,c, Video SI8). Additionally, these experiments further reveal how SPY650‐FastAct fails to efficiently label leading edge actin populations. Finally, it is always a concern that probes used to visualize actin will alter the properties of the structures they recognize. To determine if SiR‐XActin altered rapid actin dynamics in CAD cells, we measured the rate of retrograde flow at the leading edge, which is directly proportional to the rate of actin assembly.^[^ ^53^ ^]^ There was no difference in cells where actin was labeled with SiR‐XActin or LifeAct‐GFP (Figure 5d,e), and the retrograde flow rates measured with either probe were consistent with previously published results.^[^ ^49^, ^50^, ^51^ ^]^ In summary, SiR‐XActin provided superior results at labeling leading‐edge actin structures in comparison to SPY‐650‐FastAct, and did not appear to alter the behavior of the structures that it recognized (Figure 5, Videos SI7 and SI8).

## Conclusion

We have developed a new family of synthetic fluorescent probes for imaging F‐actin in live cells that distinguish themselves through their low cytotoxicity and efficient labeling in different cell types. As judged by live‐cell imaging, labeling of F‐actin with SiR‐XActin is possible without major changes in the dynamics of actin polymerization and depolymerization. The new probe is compatible with (super‐resolution) microscopy and the generation of differently colored XActin derivatives for live‐cell imaging is straightforward. SiR‐XActin and derivatives of XActin in general should become popular tools for studying the role of actin in various biological processes.

## Author Contributions

V.N., H.‐D.A., K.J. designed XActin probes and oversaw this study. V.N., J.K., K.J. designed and interpreted the experiments for profiling the properties of conjugates. V.N. synthesized all molecules reported in this study. M.B. performed cell viability experiments. J.K. performed STED imaging and photophysical characterization of conjugates. V.N., J.K., B.K, performed CLSM and widefield experiments for profiling the properties of conjugates. V. L‐D., E. A. C‐A., M. M., K. W. performed wound healing experiments. H. L., E.A.V. designed and performed labeling actin dynamic structures in live CAD cells. R.D. performed the experiments regarding cytokinesis, which were designed and interpreted by R.D. and A.E. V.N., K.J. wrote the manuscript with input from all authors.

## Conflict of Interests

KJ is inventor of patents on fluorophores filed by the Max Planck Society and Ecole Polytechnique Federale de Lausanne, which are licensed by Spirochrome. Fluorescent conjugates of XActin ligand will be distributed by Spirochrome under the name FastAct_X. Other authors declare no competing interests.

## Supporting information



Supporting information

Supporting information

Supporting information

Supporting information

Supporting information

Supporting information

Supporting information

Supporting information

Supporting information

## Data Availability

The data that support the findings of this study are available from the corresponding author upon reasonable request.
